# Supplementary figures and images for: Makorin 1 controls embryonic patterning by alleviating Bruno1-mediated repression of oskar translation
Source: PLoS Genet. 2020 Jan 24;16(1):e1008581. doi: 10.1371/journal.pgen.1008581 (PMC7001992; doi:10.1371/journal.pgen.1008581)

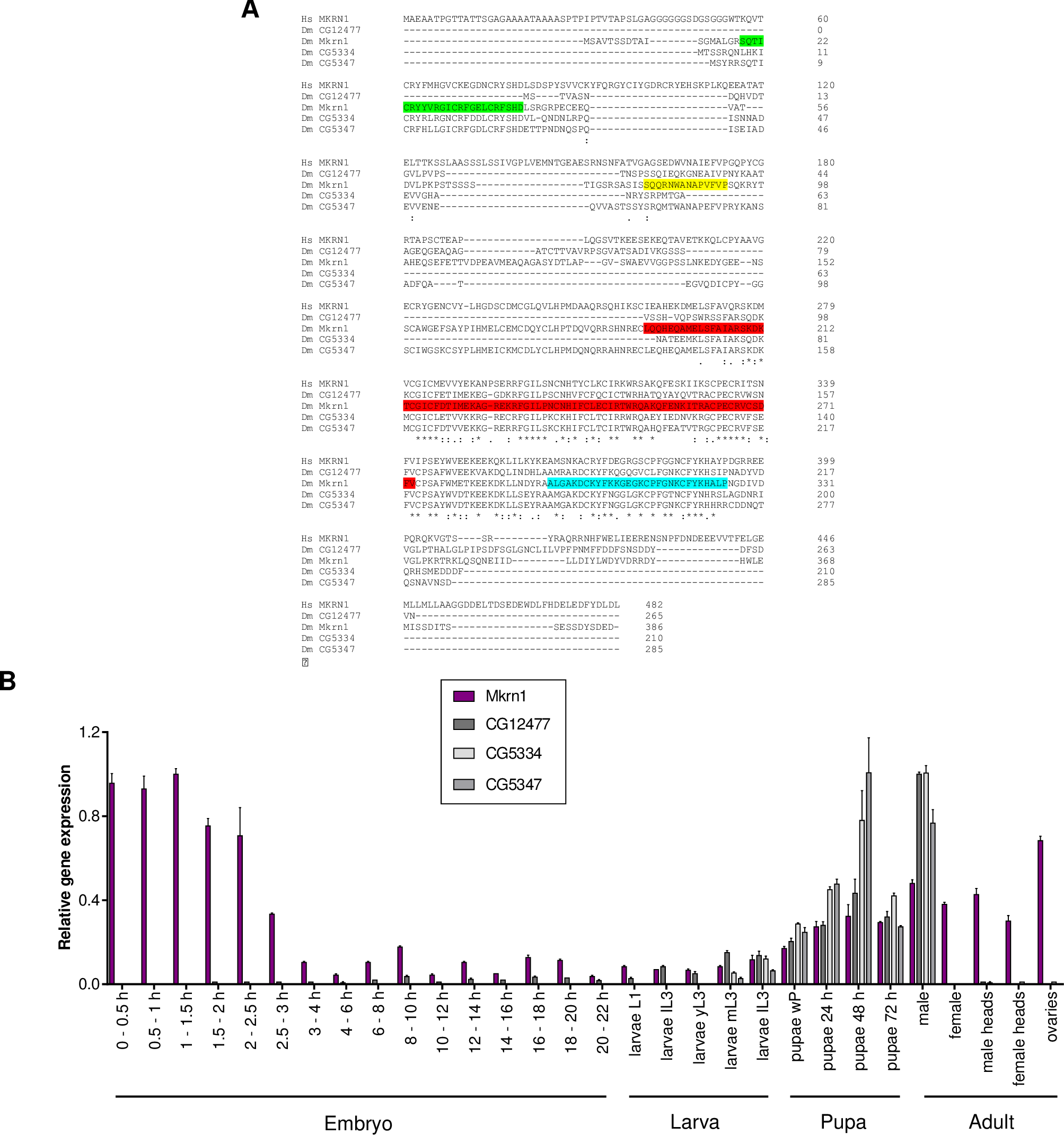

Supplement: S1 Fig — (A) Sequence alignment of human MKRN1 and the four Makorin-related proteins in Drosophila, Mkrn1, CG5334, CG5347, and CG12477. The ZnF1 domain in Mkrn1 is highlighted green, the PAM2 motif is highlighted yellow, the RING domain is highlighted red, and the ZnF2 domain is highlighted light blue. The RING and ZnF2 domains are conserved in all four proteins, whereas the PAM2 motif is only conserved in CG12477 and CG5347, and ZnF1 is conserved in CG5334 and CG5347. (B) Relative mRNA levels of Mkrn1 and the three other genes encoding predicted Makorin proteins at various stages of development, as measured by RT-qPCR. mRNA levels were normalized to Rpl15 mRNA. Error bars depict Stdev, n = 3. (TIF) [file pgen.1008581.s001.tif]

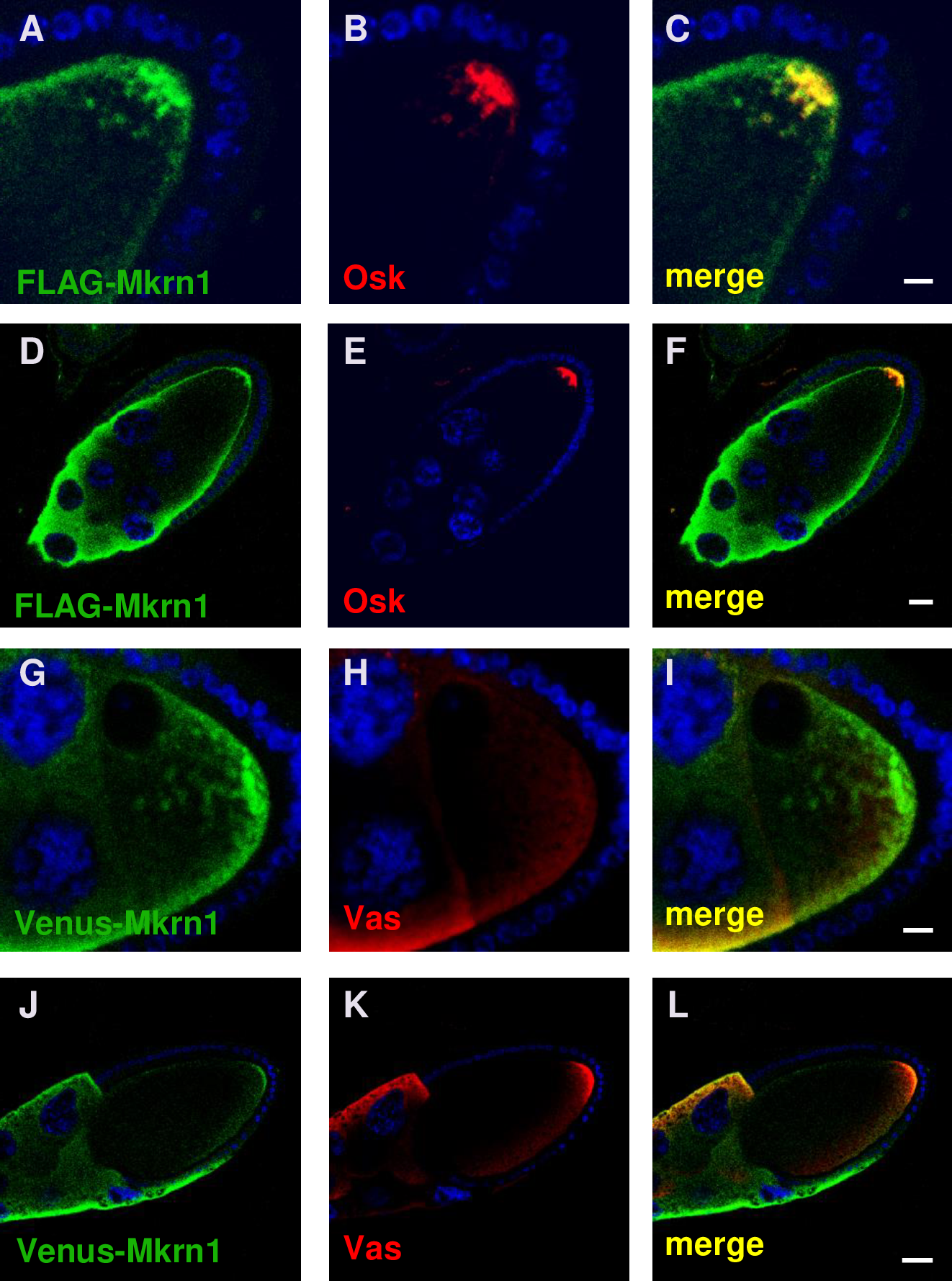

Supplement: S2 Fig — All images are from wild-type oocytes expressing Venus-Mkrn1 or FLAG-Mkrn1 as indicated. Overexpression was performed using a nos>Gal4 driver. (A, D,) Immunostaining with α-FLAG to monitor FLAG-Mkrn1. (G and J) Immunostaining with α-GFP recognizing Venus-Mkrn1. (B and E) Immunostaining with α-Osk. (H and K). (C, F, I, L,) Merged images from two preceding panels. Scale bars: (A-C, G-I,) 5 μm; (D-F, J-L) 20 μm. (TIF) [file pgen.1008581.s002.tif]

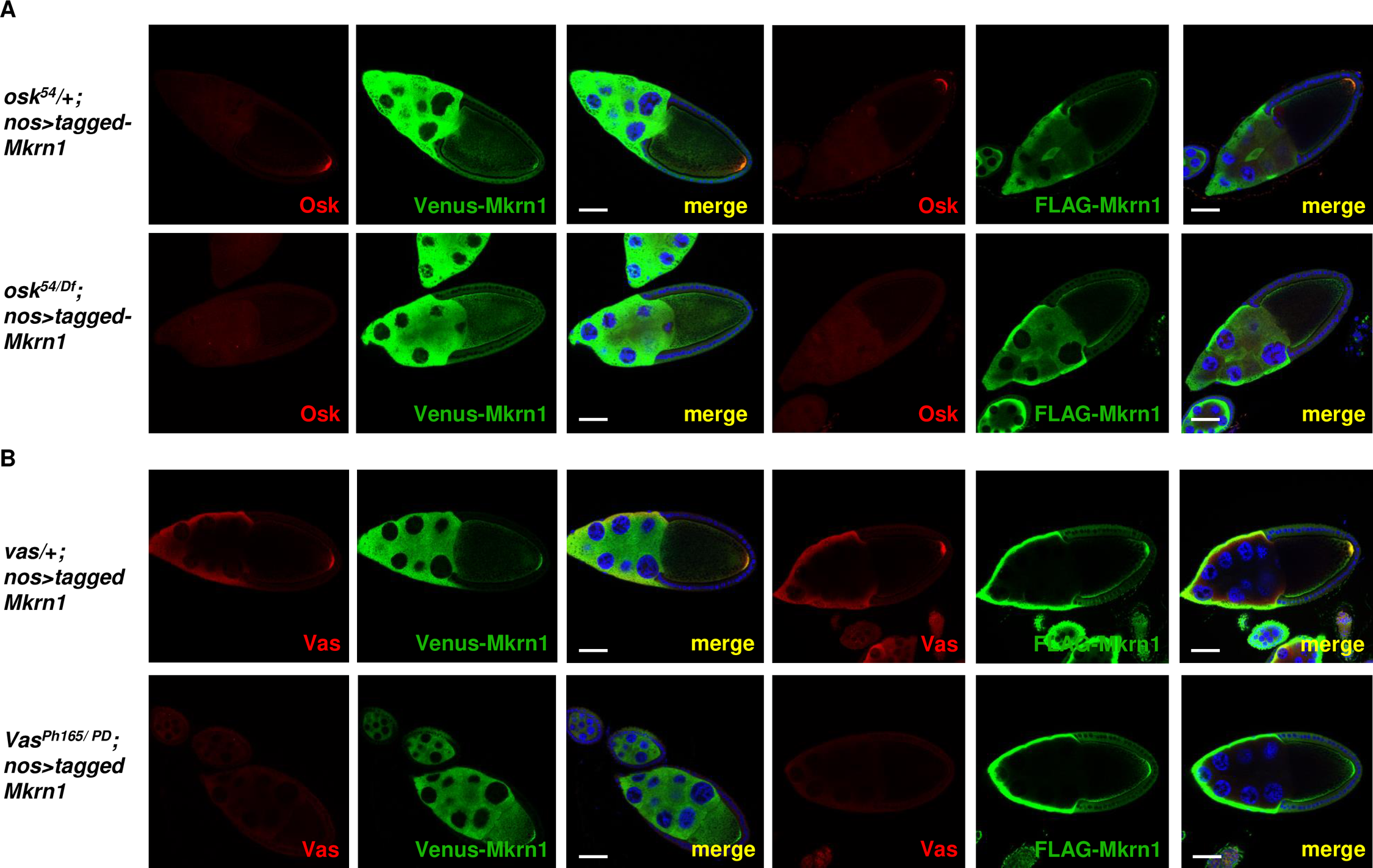

Supplement: S3 Fig — (A) Posterior accumulation of either Venus-Mkrn1 or FLAG-Mkrn1 is normal in osk54/+ oocytes but is absent in osk54/Df(3R)p-XT103 (osk) oocytes. (B) Posterior accumulation of either Venus-Mkrn1 or FLAG-Mkrn1 is normal in both vas1/+ or vas1/vasPH (vas) oocytes. Scale bars, 50 μm. (TIF) [file pgen.1008581.s003.tif]

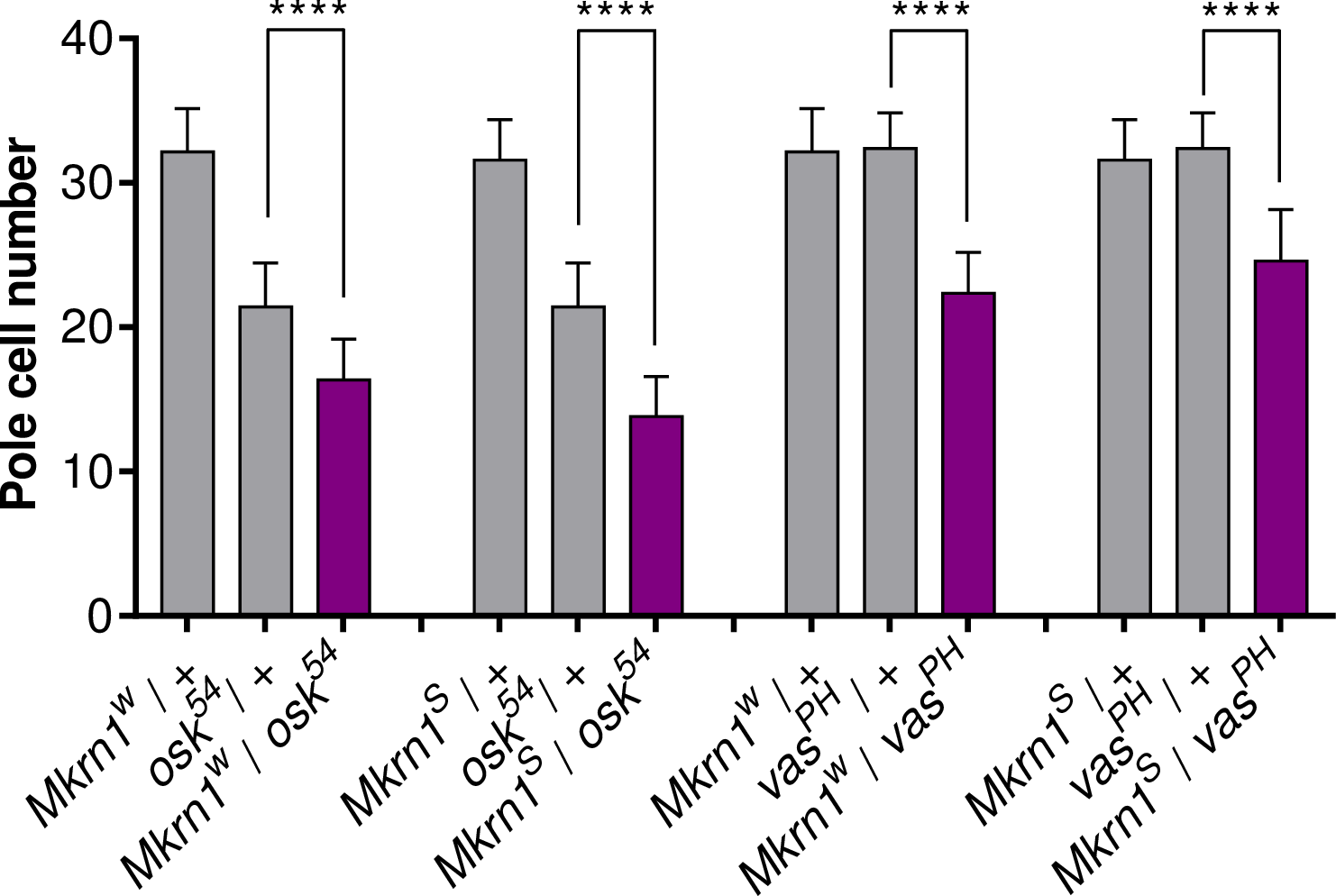

Supplement: S4 Fig — Pole cell counts from embryos produced by females with the indicated genotypes. Embryos from trans-heterozygotes for Mkrn1 and osk or vas mutations have fewer pole cells than those from single heterozygote controls. Error bars illustrate Stdev, n = 60. (TIF) [file pgen.1008581.s004.tif]

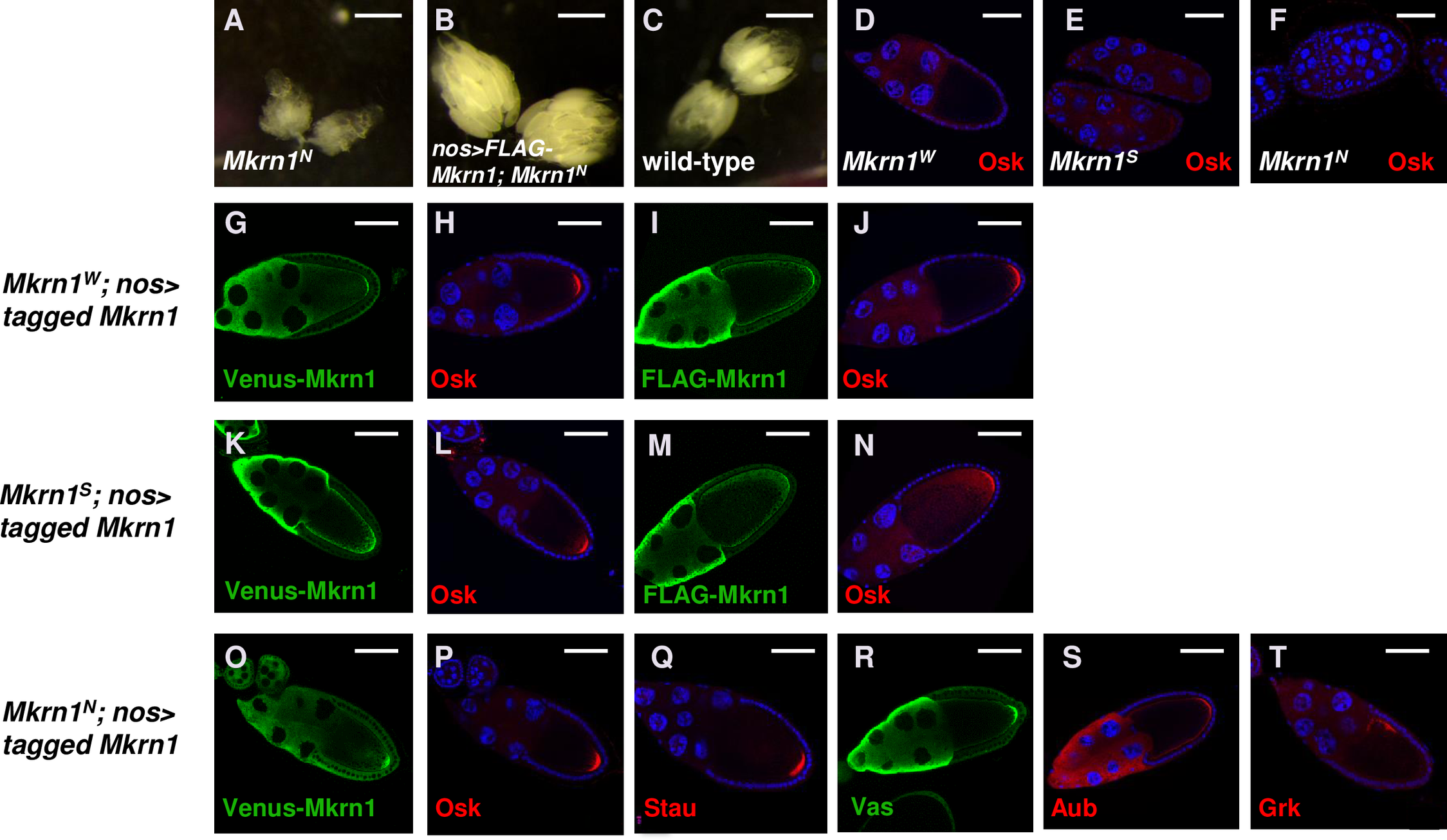

Supplement: S5 Fig — (A-C) Bright-field micrographs of entire ovaries from (A) Mkrn1N; (B) nos>FLAG-Mkrn1; Mkrn1N and (C) wild-type females, showing overall rescue of oogenesis. Scale bars, 500 μm. (D-F) α-Osk immunostaining on (D) Mkrn1W, (E) Mkrn1S, (F) Mkrn1N egg chambers as negative controls. (G-J) Transgenic expression of tagged Mkrn1 restores posterior localization of Osk protein in Mkrn1W oocytes. (G, H) nos>Venus-Mkrn1; Mkrn1W; (I and J) nos>FLAG-Mkrn1; Mkrn1W. (H and J) Immunostaining with α -Osk; (G) Immunostaining with α-GFP to visualize Venus-Mkrn1; (I) Immunostaining with α-FLAG recognizing FLAG-Mkrn1. (K-N) Transgenic expression of tagged Mkrn1 restores expression and posterior localization of Osk protein in Mkrn1S oocytes. (K and L) nos>Venus-Mkrn1; Mkrn1S; (M and N) nos>FLAG-Mkrn1; Mkrn1S. (L and N) Immunostaining using α-Osk; (K) Immunostaining with α-GFP recognizing Venus-Mkrn1; (M) Immunostaining using α-FLAG to visualize FLAG-Mkrn1. (O and P) Transgenic expression of tagged Mkrn1 restores expression and posterior localization of Osk protein in Mkrn1N oocytes. (Q-T) Immunostaining experiments revealing localization of various proteins in nos>Venus-Mkrn1; Mkrn1N oocytes. (Q) α -Stau; (R) α -Vas; (S) α -Aub; (T) α -Grk. (D-T) Scale bars, 50 μm. (TIF) [file pgen.1008581.s005.tif]

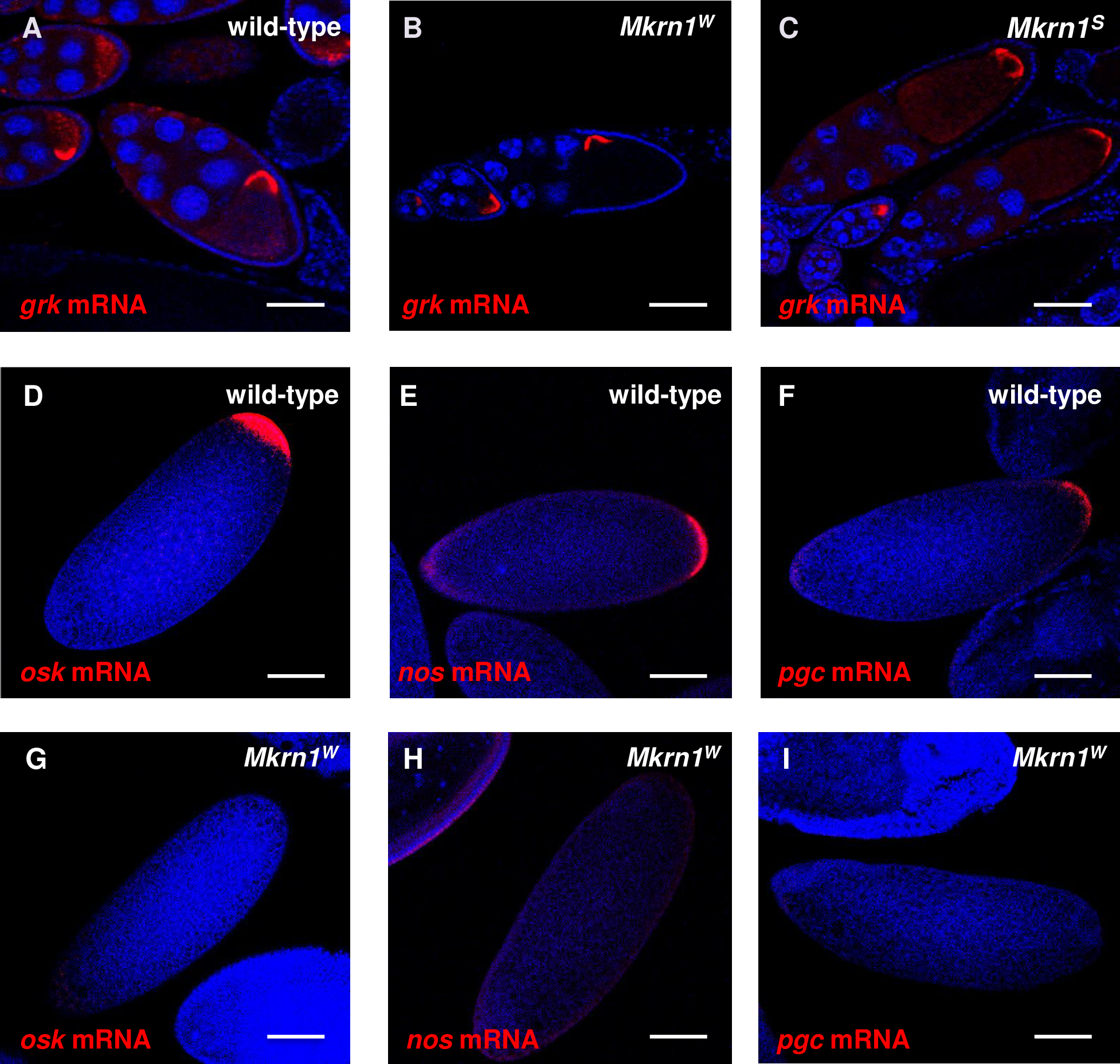

Supplement: S6 Fig — (A and B) Antero-dorsal accumulation of grk mRNA is similar to wild-type in stage 10 Mkrn1W oocytes. Scale bars, 50 μm. (C) grk mRNA remains associated with the oocyte nucleus and is mislocalized to the posterior in stage 10 Mkrn1S oocytes. Scale bars, 50 μm. In situ hybridization experiments showing posterior accumulation of (D) osk, (E) nos, and (F) pgc mRNAs in wild-type embryos. Scale bars, 100 μm. (G-I) Posterior accumulation of these mRNAs is lost in Mkrn1W embryos. Scale bars, 100 μm. (TIF) [file pgen.1008581.s006.tif]

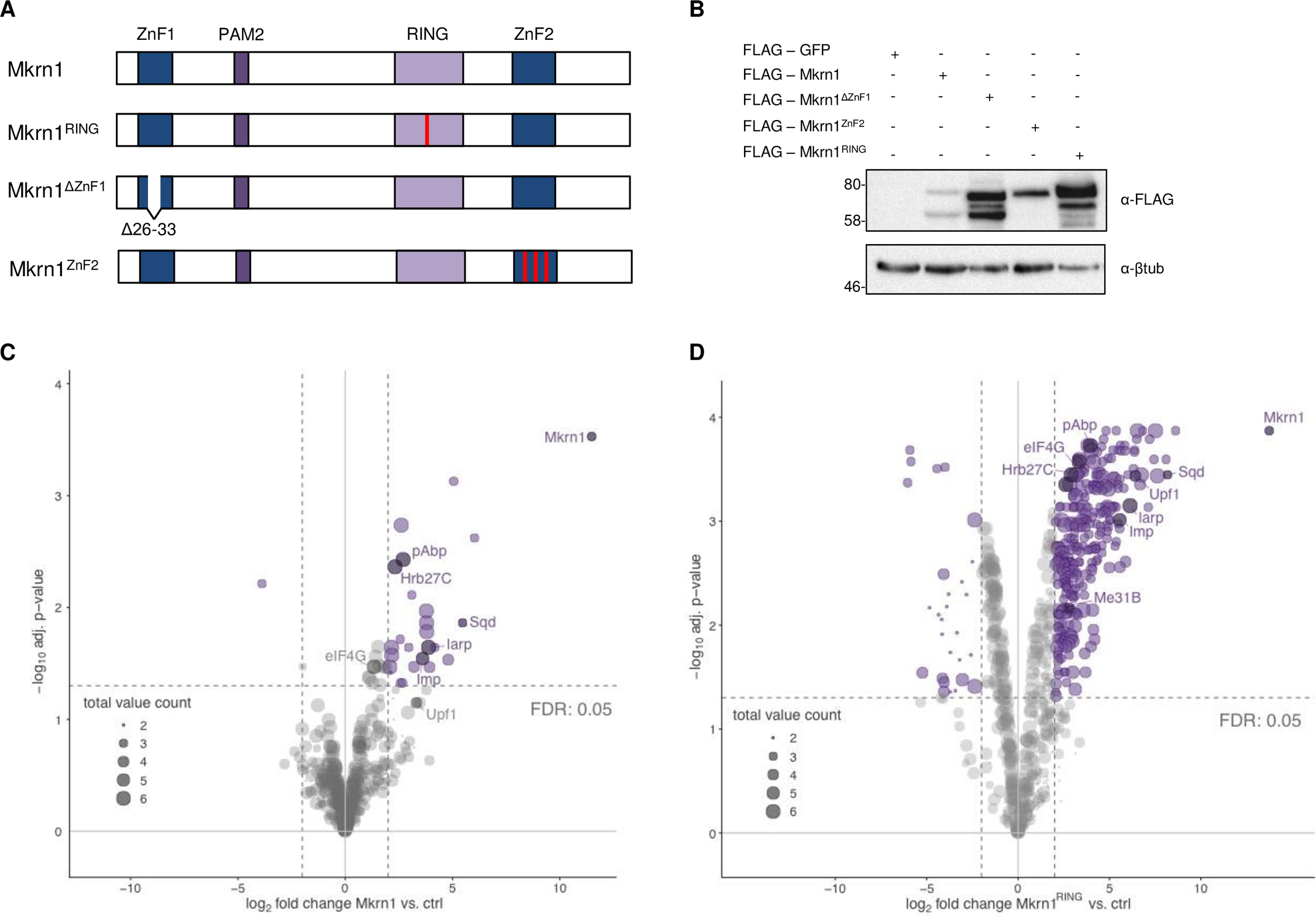

Supplement: S7 Fig — (A) Schematic diagram of Mkrn1 constructs with functional domains highlighted. Differenet mutations were introduced into Mkrn1 protein: Mkrn1RING carries a point mutation that changes histidine 239 to glutamic acid (H239E) while Mkrn1ΔZnF1 contains a deletion of amino acids 26 to 33. To disrupt the ZnF2 domain (Mkrn1ZnF2) three point mutations that mutate cysteines to alanines at positions 302, 312 and 318 (C302A, C312A and C318A) were introduced. (B) Immunoblot showing the relative expression levels of various forms of FLAG-Mkrn1 in S2R+ cells. (C, D) Volcano plots showing the interactome of (C) Myc-Mkrn1 and (D) Myc-Mkrn1RING in S2R+ cells identified using LC-MS/MS and label-free quantification. For both experiments, 3 technical replicates of Myc-GFP (ctrl) and Myc-Mkrn1 IP were performed and compared with each other. The enrichment of proteins compared to the control was plotted in a volcano plot using a combined cutoff of log2 fold change ≥ 2 and an FDR ≤ 0.05. Several proteins of interest are labelled. The entire list of enriched proteins can be found in S1 and S2 Tables. (TIF) [file pgen.1008581.s007.tif]

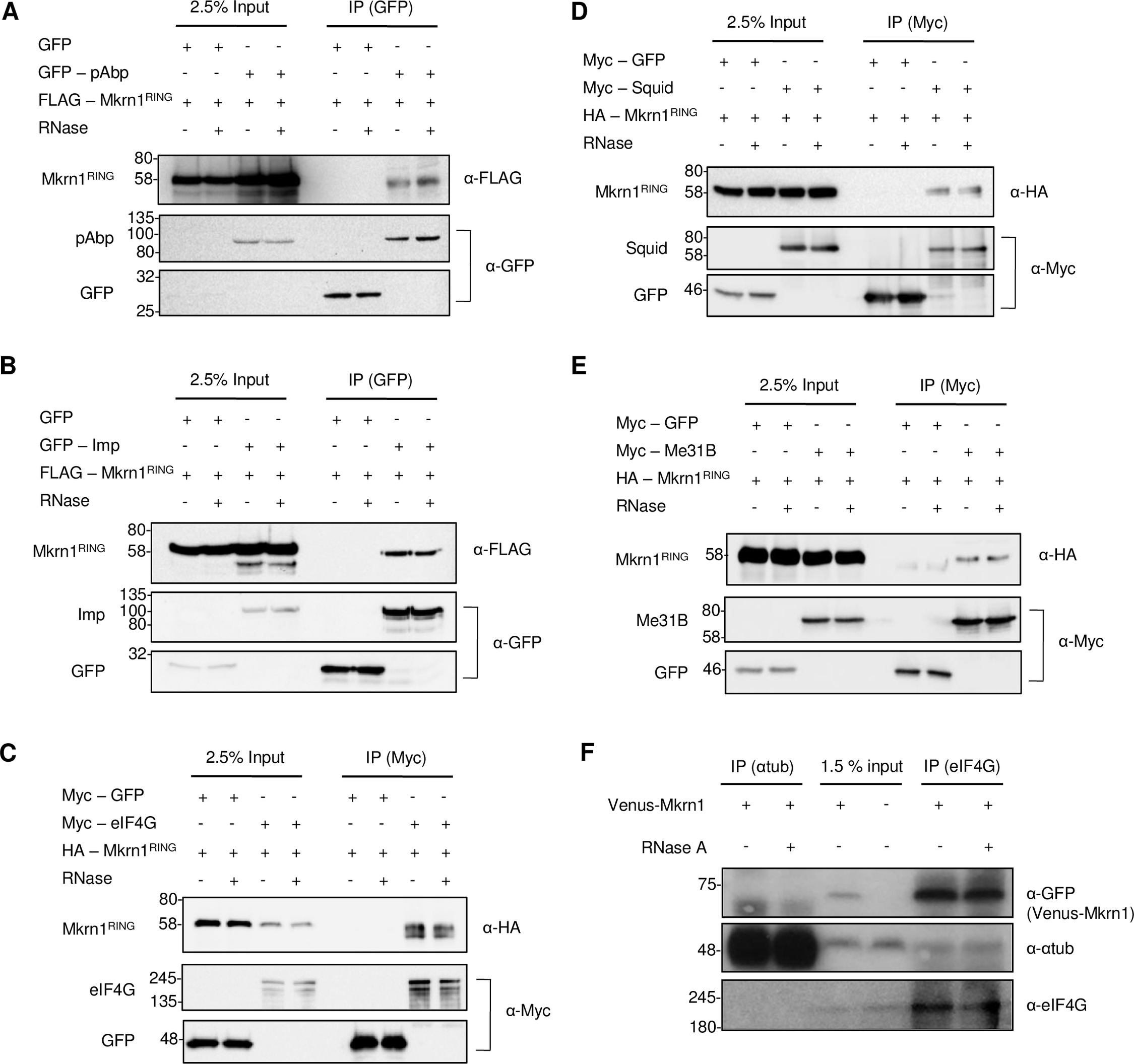

Supplement: S8 Fig — Pulldown experiments to validate binding of tagged Mkrn1RING with (A) GFP-pAbp, (B) GFP-Imp, (C) Myc-eIF4G (D) Myc-Sqd and (E) Myc-Me31B. GFP and Myc IPs were performed in the absence or presence of RNase T1 and enrichment of the proteins was analyzed by immunoblotting. As controls, either GFP alone or Myc-GFP were used. All co-IP experiments were performed in S2R+ cells. (F) Western blot depicting co-IP experiments between Venus-Mkrn1 and eIF4G in ovaries. α-tubulin (αtub, lanes 1, 2) and ovaries lacking the Venus-Mkrn1 transgene (lane 4) were used as negative controls. (TIF) [file pgen.1008581.s008.tif]

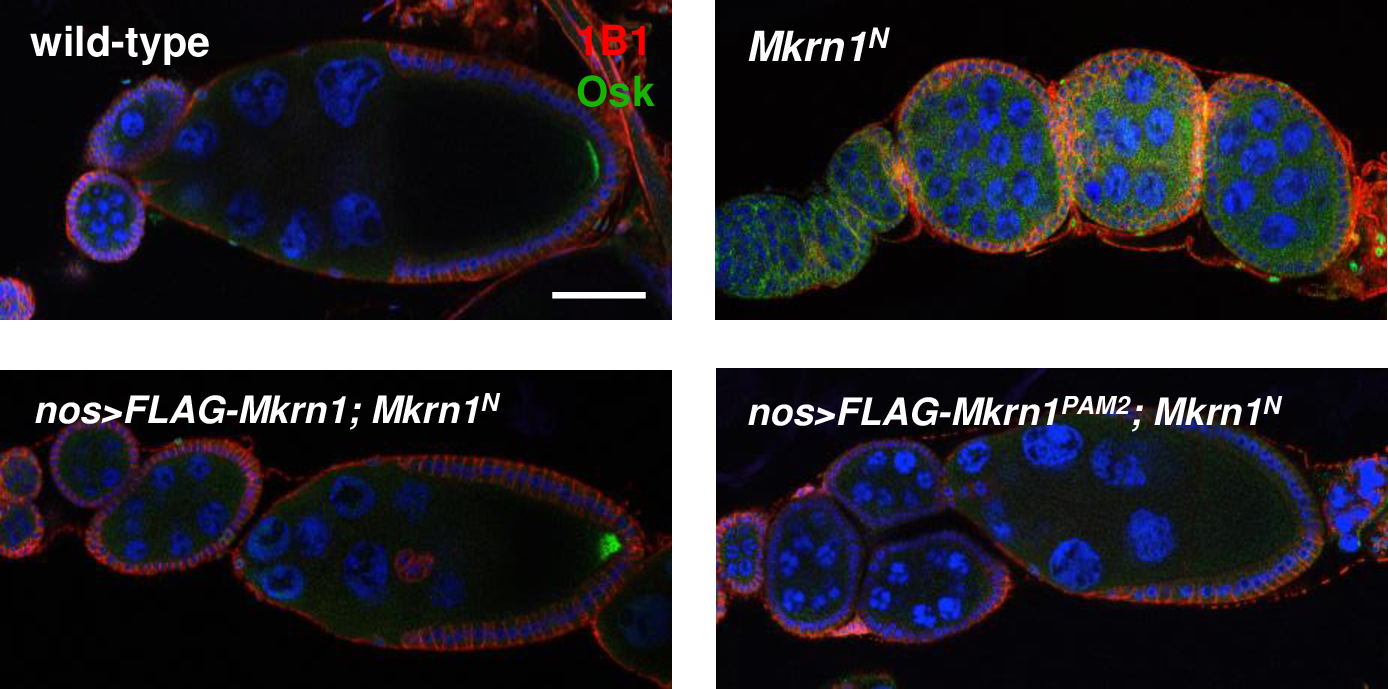

Supplement: S9 Fig — Rescue experiments of either Mkrn1 or Mkrn1PAM2 in Mkrn1N ovaries. FLAG-tagged Mkrn1 transgenes were overexpressed in ovaries using a nos>Gal4 driver line. Ovaries were stained with α-1B1 (red) and α-Osk (green). Nuclei were stained using DAPI (blue). Although overexpression of wild-type Mkrn1 could restore Osk protein at the posterior, Mkrn1PAM2 could not. Scale bar, 50 μm. (TIF) [file pgen.1008581.s009.tif]

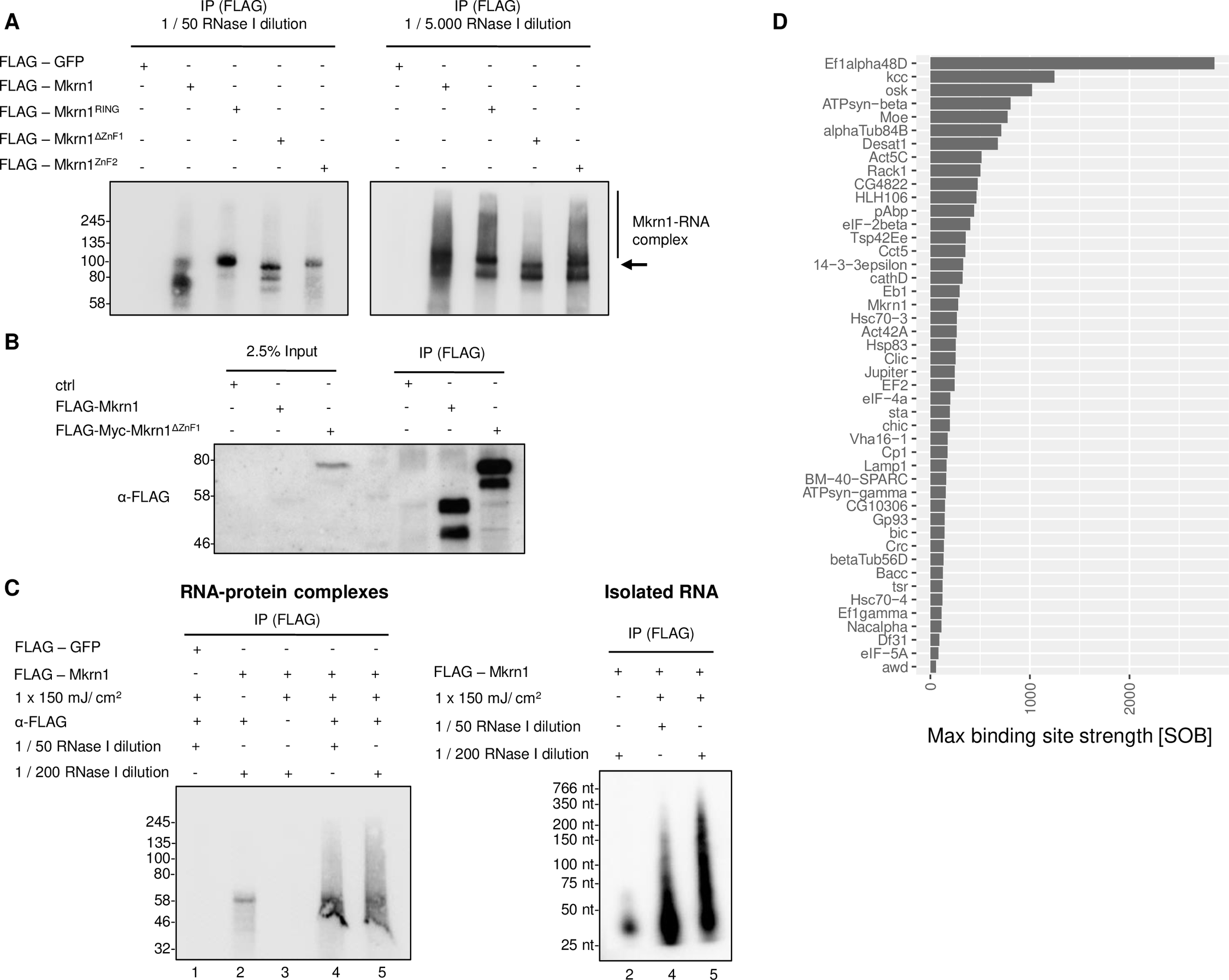

Supplement: S10 Fig — (A) The RNA binding activity of Mkrn1 is mediated by its ZnF1 domain. Autoradiographs showing association of various forms of Mkrn1 to RNA. FLAG-tagged GFP was used as a negative control. Crosslinked RNA-protein complexes were immunoprecipitated with α-FLAG and treated with different dilutions of RNase I (left: 1/50, right: 1/5000). RNA was subsequently radiolabelled and the RNA-protein complexes were separated by SDS-PAGE. Bound RNA of different sizes is detected by a smear extending upward from the sharp bands that correspond to the sizes of the FLAG-Mkrn1 proteins (arrow). (B) Representative immunoblot of RIP experiment shown in Fig 6A. Either Mkrn1 or Mkrn1ΔZnF1 were overexpressed in Mkrn1N ovaries using a nos>Gal4 driver. The proteins were immunoprecipitated using α-FLAG antibody. Note that Mkrn1ΔZnF1 protein runs higher because of the presence of an additional Myc tag. (C) Validation of iCLIP experiments. Immunoprecipitation of FLAG-Mkrn1 was performed in different conditions. S2R+ cells were transfected and UV-crosslinked prior to IP experiments. Left: autoradiograph showing protein-RNA complexes. Right: Signals of lanes 2, 4 and 5 in autoradiograph were cut and RNAwas subsequently isolated. RNA length was analyzed on a TBE-urea gel. (D) iCLIP datasets with Mkrn1-FLAG in S2R+ cells. The x axis displays maximum binding strength per gene (SOB) and the y axis shows the gene identity. The genes are sorted by SOB with osk mRNA appearing at the third place. Note that ribosomal genes have been excluded for clarity. (TIF) [file pgen.1008581.s010.tif]

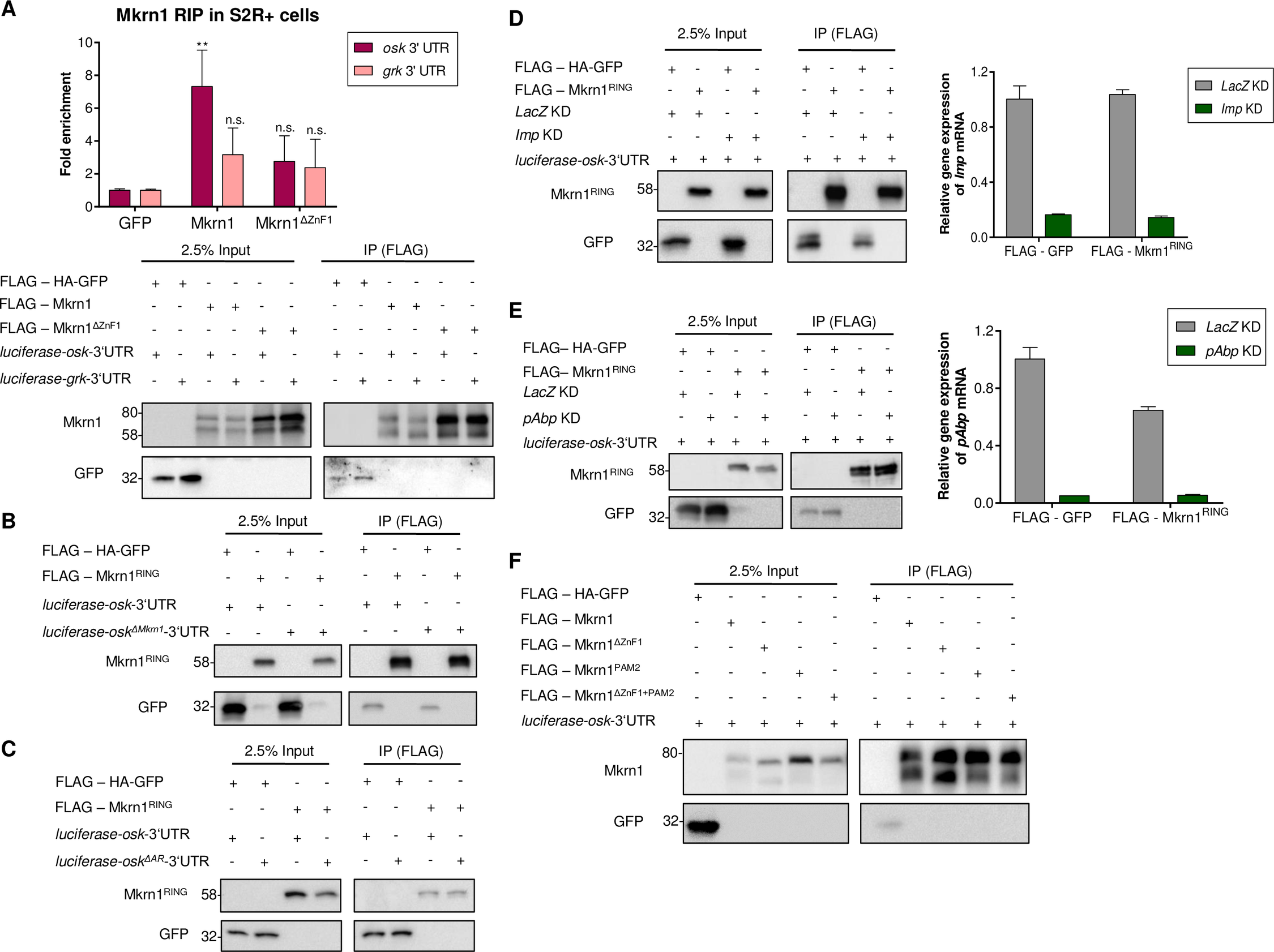

Supplement: S11 Fig — (A) Mkrn1, but not Mkrn1ΔZnF1 binds to the osk 3’ UTR in S2R+ cells. FLAG-RIP of GFP, Mkrn1 and Mkrn1ΔZnF1 was performed co-expressing either luciferase-grk-3’UTR or luciferase-osk-3’UTR reporter. Top: qPCR analysis of the RIP experiments. Fold enrichment is illustrated relative to GFP RIP. Error bars depict SEM, n = 3. Bottom: Immunoblots of a representative RIP experiment. (B-F) Western blot analysis of a representative FLAG-RIP experiment in S2R+ cells summarized in Fig 6C–6F. (B) Either FLAG-tagged GFP or Mkrn1RING was overexpressed. RIP experiments were performed in the presence of a luciferase-osk-3’UTR reporter containing osk 3’ UTR wild-type sequence or deletion of the Mkrn1 binding site (oskΔMkrn1) (C) FLAG-RIP of GFP or Mkrn1RING were performed either using the luciferase-osk-3’UTR reporter. Binding was compared between wild-type osk 3’ UTR and a mutation in the AR region (oskΔAR). (D and E) RIP experiments against FLAG-Mkrn1RING were performed in control (LacZ) condition and compared to (D) Imp or (E) pAbp mRNA knockdown. Right: RT-qPCR analysis of the knockdown efficiency. Imp and pAbp mRNA levels were normalized to Rpl15 mRNA. (F) FLAG-RIP experiments in S2R+ cells using different Mkrn1 mutants. Representative immunoblot is depicted. (TIF) [file pgen.1008581.s011.tif]

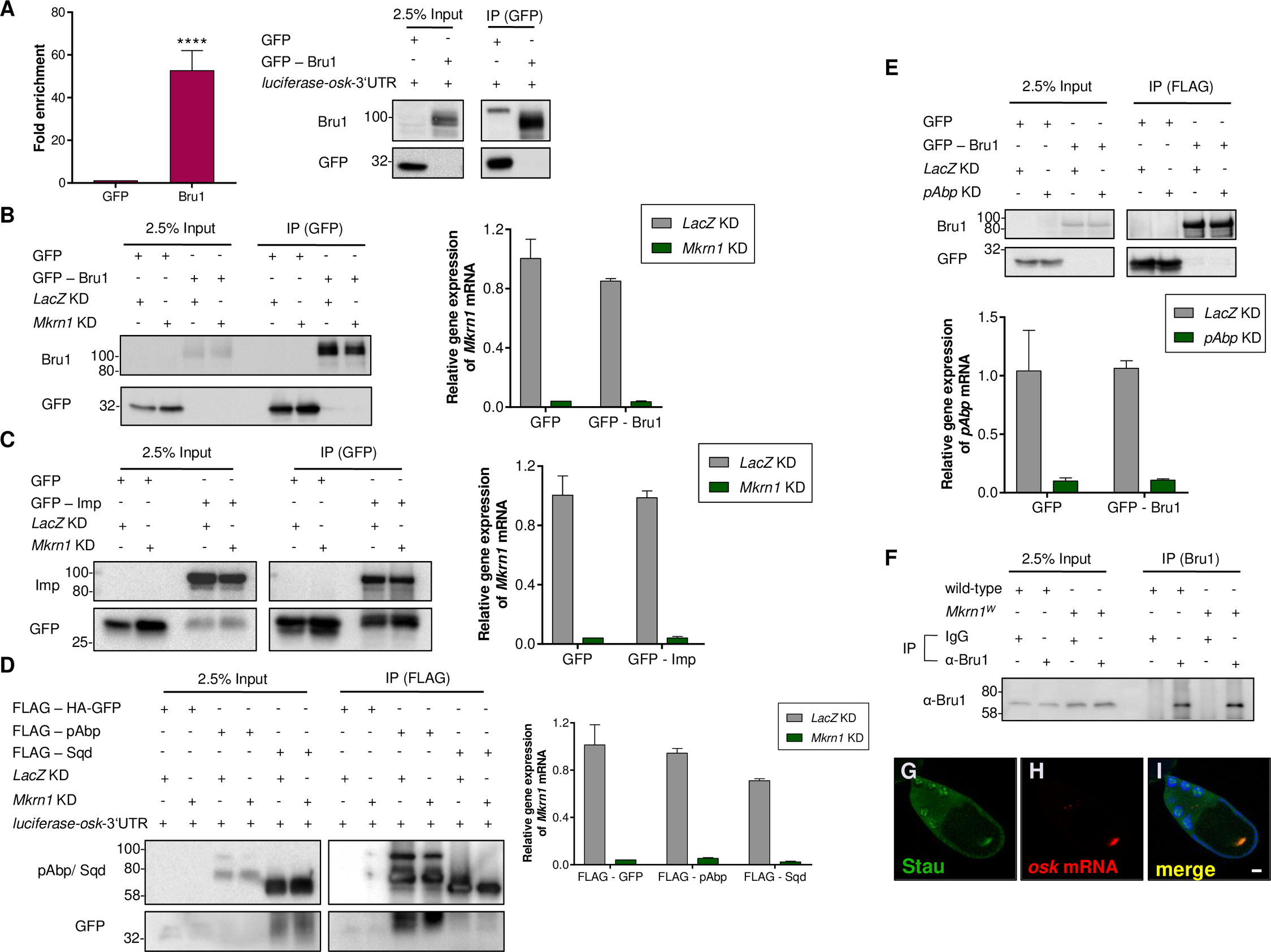

Supplement: S12 Fig — (A) RIP experiments of GFP alone or GFP-Bru1 in S2R+ cells. Left: qPCR analysis of RIP experiments analyzing the enrichment of the luciferase-osk-3’UTR transcript. Error bars depict SEM n = 3. Right: Immunoblot of one representative IP using α-GFP. (B-F) Immunoblots of representative RIP experiments summarized in Fig 7A–7C. Right: RT-qPCR validation of the respective knockdown. mRNA levels were normalized to Rpl15 mRNA. (B) RIP experiment of either GFP alone or GFP-Bru1 were performed in control (LacZ) or Mkrn1 mRNA knockdown condition. (C) GFP-RIP experiments were performed in control (LacZ) or Mkrn1 mRNA knockdown condition for either GFP or GFP-Imp. (D) RIP experiment performed against FLAG-tagged pAbp or Sqd in LacZ or Mkrn1 mRNA depleted cells. As control, RIP was performed with FLAG-tagged GFP. (E) GFP-RIP of either GFP alone or GFP-Bru1 in control (LacZ) or pAbp KD. (F) Representative immunoblot of a RIP experiment using heterozygous (wild-type) or homozygous Mkrn1W ovary lysate against endogenous Bru1. As control RIP, normal rabbit IgG was used. (G-I) The three panels show the same Mkrn1W stage 10 egg chamber stained for (G) Stau, (H) osk mRNA and (I) a merged image. There is accumulation of Stau near the pole plasm and co-localization with osk mRNA. Scale bar, 20 μm. (TIF) [file pgen.1008581.s012.tif]

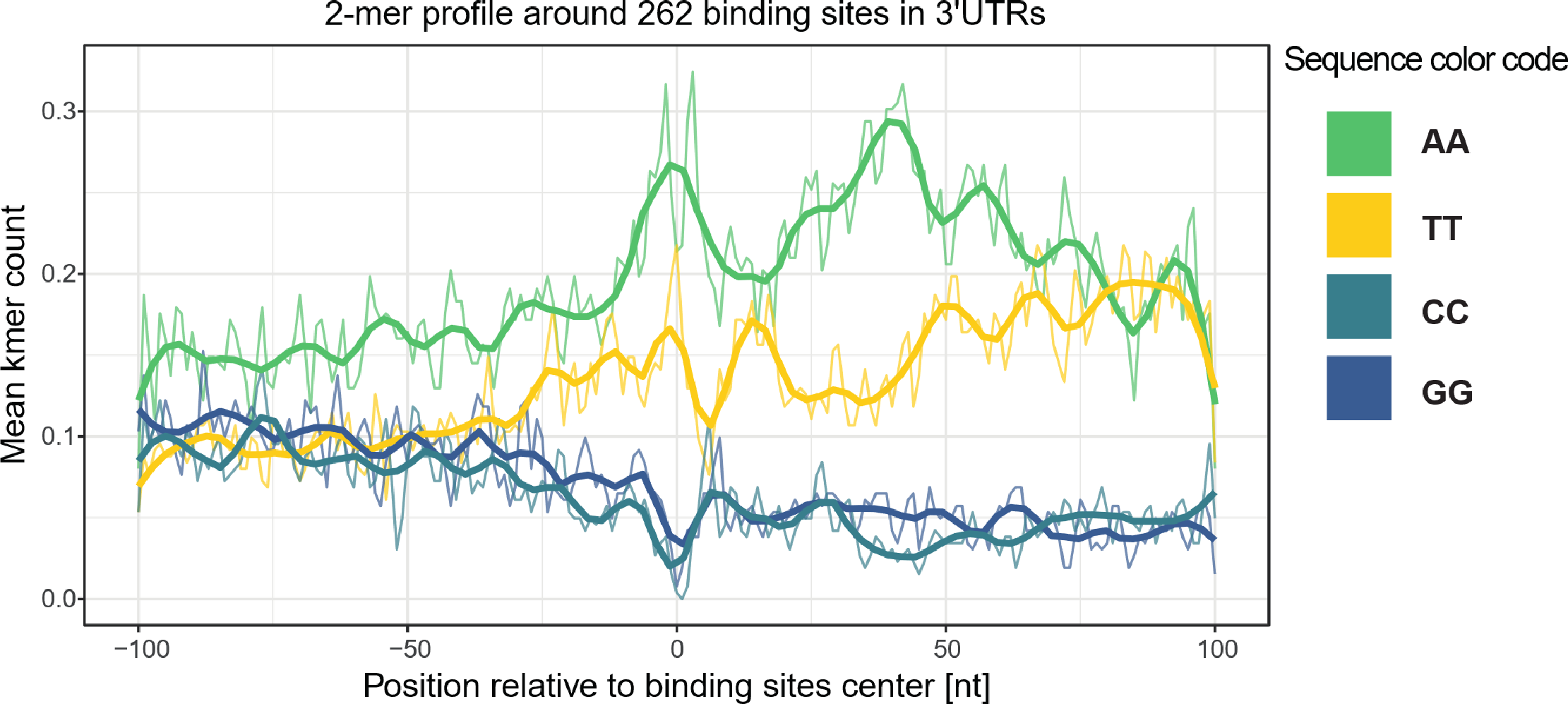

Supplement: S13 Fig — Homopolymeric dimers were counted in 200 a nucleotide wide window centered at 262 binding sites midpoints in 3’ UTRs. (TIF) [file pgen.1008581.s013.tif]
