# Supplementary material for: Makorin 1 controls embryonic patterning by alleviating Bruno1-mediated repression of oskar translation
Source: PLoS Genet. 2020 Jan 24;16(1):e1008581. doi: 10.1371/journal.pgen.1008581 (PMC7001992; doi:10.1371/journal.pgen.1008581)
Supplement: S3 Table — (DOCX) [file pgen.1008581.s016.docx]

**S3 Table.** List of gRNAs used in this study and primers used to validate mutations of the *Mkrn1* gene by CRISPR/ Cas9.

| **Name** | **Sequence** |
| --- | --- |
| gRNA1 | GTCGATCTGCCGCTACTACGTGCG |
| gRNA2 | GTCGCAGCTACGATACATTAGCCC |
| gRNA3 | CTTCGAATCTGCGTATGTACGCAG |
| gRNA4 | CTTCGACTAAGCTTCTGAAGCAGA |
| Mkrn1_gDNA_F | CGAACAACTGTGGTTTAAGGGT |
| Mkrn1_gDNA_R2 | GGATTGGTGTGTGCGTTTCA |
