# Supplementary material for: Makorin 1 controls embryonic patterning by alleviating Bruno1-mediated repression of oskar translation
Source: PLoS Genet. 2020 Jan 24;16(1):e1008581. doi: 10.1371/journal.pgen.1008581 (PMC7001992; doi:10.1371/journal.pgen.1008581)
Supplement: S4 Table — (DOCX) [file pgen.1008581.s017.docx]

**S4** **Table.** List of primers used to create PCR templates of dsRNAs.

| **Name** | **Sequence** |
| --- | --- |
| RNAi-LacZ-F | TAATACGACTCACTATAGGGCAGGCTTTCTTTCACAGATG |
| RNAi-LacZ-R | TAATACGACTCACTATAGCTGATGTTGAACTGGAAGTC |
| RNAi_Mkrn1_F | TAATACGACTCACTATAGGGTCTCCAGTCAGCAGAGGAAC |
| RNAi_Mkrn1_R | TAATACGACTCACTATAGCGTTGAGCAATTTGTCCTTT |
| RNAi_PABPC1_F | TAATACGACTCACTATAGGGTCAGGCTCTCAATGGCAAGG |
| RNAi_PABPC1_R | TAATACGACTCACTATAGTGATTTGACGGAAGGGTCGG |
| RNAi_IMP_F | TAATACGACTCACTATAGGGGGTCTGAACGGTGTCGAGTT |
| RNAi_IMP_R | TAATACGACTCACTATAGGTGTCGACATCATTGCCATC |
