# Supplementary material for: Makorin 1 controls embryonic patterning by alleviating Bruno1-mediated repression of oskar translation
Source: PLoS Genet. 2020 Jan 24;16(1):e1008581. doi: 10.1371/journal.pgen.1008581 (PMC7001992; doi:10.1371/journal.pgen.1008581)
Supplement: S5 Table — (DOCX) [file pgen.1008581.s018.docx]

**S5** **Table.** List of primers used to in qPCR experiments.

| **Name** | **Sequence** |
| --- | --- |
| rpl15_F | AGGATGCACTTATGGCAAGC |
| rpl15_R | GCGCAATCCAATACGAGTTC |
| Mkrn1_mRNA_qPCR_F2 | CCCAACGGAGATATCGTCGA |
| Mkrn1_mRNA_qPCR_R2 | AGCTTAGTCTTCATCCGAGTAATCT |
| CG12477_mRNA_qPCR_F1 | AACCAATCCAATAGAGCAG |
| CG12477_mRNA_qPCR_R1 | TTCACTATGTCGGCAGAG |
| CG5334_mRNA_qPCR_F2 | GTTGCTAAGCGAATATCGCG |
| CG5334_mRNA_qPCR_R2 | TAAAAGTCGTCATCCTCCATTG |
| CG5347_mRNA_qPCR_F2 | CGTGAACGTCGCTTTGGCAT |
| CG5347_mRNA_qPCR_R2 | CCCATTGCCGCGCGATATTC |
| osk_mRNA_F2 | TTCGCTTGCACAAAATCAAC |
| osk_mRNA_R2 | TTTGCAAACGGAAACAGAAA |
| grk_mRNA_F2 | AGCTTTCGTTGGAGCTTTTG |
| grk_mRNA_R2 | TCGAGTCCCAATCCTCTTCT |
| bcd_mRNA_F2 | AACATTTGCGCATTCTTTGA |
| bcd_mRNA_R2 | AGTTATTCCGTTTGGCAGCA |
| 18S_F | CTGAGAAACGGCTACCACATC |
| 18S_R | ACCAGACTTGCCCTCCAAT |
| Fluc_F | CCAGGGATTTCAGTCGATGT |
| Fluc_R | AATCTGACGCAGGCAGTTCT |
| dPABP_cDNA_F | CCAGCAGCGTACTTCCAACT |
| dPABP_cDNA_R | CCCAGGATCTGTTTCTGCTC |
| Imp_mRNA_F | TCCGGTGGAGATGAAGAGAC |
| Imp_mRNA_R | TGTTCTTTGGCAGCTTTCTG |
