# Supplementary material for: Makorin 1 controls embryonic patterning by alleviating Bruno1-mediated repression of oskar translation
Source: PLoS Genet. 2020 Jan 24;16(1):e1008581. doi: 10.1371/journal.pgen.1008581 (PMC7001992; doi:10.1371/journal.pgen.1008581)
Supplement: S6 Table — (DOCX) [file pgen.1008581.s019.docx]

**S6 Table.** List of barcodes used to prepare iCLIP libraries from material of S2R+ cells

| **Name** | **Barcode Sequence** |
| --- | --- |
| Control | NNNGGTCNN |
| Rep1 | NNNTTAANN |
| Rep2 | NNNATACNN |
| Rep3 | NNNTACGNN |
| Rep4 | NNNGAAANN |
| Rep5 | NNNATCGNN |
| Rep6 | NNNCGAGNN |
