# Supplementary material for: Makorin 1 controls embryonic patterning by alleviating Bruno1-mediated repression of oskar translation
Source: PLoS Genet. 2020 Jan 24;16(1):e1008581. doi: 10.1371/journal.pgen.1008581 (PMC7001992; doi:10.1371/journal.pgen.1008581)
Supplement: S7 Table — (DOCX) [file pgen.1008581.s020.docx]

| **Name** | **Sequence** |
| --- | --- |
| Mkrn1_wt_KpnI_F | CACCGGTACCATGAGTGCCGTCACCAG |
| Mkrn1_wt_XbaI_R | TCTAGAGTCTTCATCCGAGTAATCTGA |
| dPABP_KpnI_F2 | AAAAGGTACCATGGCTTCTCTATACGTCGGTGA |
| dPABP_XbaI_R2 | AAAATCTAGAGTTGGCGGGCTCGGTGA |
| Imp_KpnI_F | AAAAGGTACCATGCACAGCAACAATAATAGCA |
| Imp_XbaI_R | AAAATCTAGACTGTTGTGAGCTCGCCAG |
| eif4G-PB_Notl_F | AAAGCGGCCGCCATGCAACAGGCTATACCAAC |
| eif4G-PB_AscI_R | AAAGGCGCGCCCGTTGGCATCATCGTTTAACA |
| me31B-RA_NotI_F | AAAGCGGCCGCCATGATGACTGAAAAGTTAAATTC |
| me31B-RA_AscI_R | AAAGGCGCGCCCTTTGCTAACGTTGCCCTC |
| Sqd_NotI_F | AAAGCGGCCGCCATGGCCGAGAACAAGCAA |
| Sqd_AscI_R | AAAGGCGCGCCCGAACTGCTGATAGTTGTTGCT |
| bruno_RA_KpnI_F | AAAAGGTACCATGTTCACCAGCCGCGCTT |
| bruno_RA_XbaI_R | AAATCTAGAGTAGGGCTTCGAGTCCTTGGG |
| Mkrn1_H239E_F | TGCAACGAAATATTCTGCTTGGAGTGCAT |
| Mkrn1_H239E_R | CAGAATATTTCGTTGCAGTTGGGGAGAATGC |
| Mkrn1_ZnF1_del_F | GGAGCCAGACCATCTGCCGCTTTGGGGAACTTTGCCGC |
| Mkrn1_ZnF1_del_R | GTTCCCCAAAGCGGCAGATGGTCTGGCTCC |
| Mkrn1_Znf2_F | AGGACGCCAAATACTTTAAAAAAGGAGAGGGCAAGGCCCCCTTTGGTAACAAGGCCTTCTACAAGCACGCTCTGCCCAA |
| Mkrn1_Znf2_R | TGTAGAAGGCCTTGTTACCAAAGGGGGCCTTGCCCTCTCCTTTTTTAAAGTATTTGGCGTCCTTTGCTCCCAAAGCGG |
| Mkrn1_PAM2_mut_F | CCCTGTGGCCGTGGCCAGCCAAAAGCGCTATACTGC |
| Mkrn1_PAM2_mut_R | TTGGCTGGCCACGGCCACAGGGGCGTTCGCCCAGT |
