# Supplementary material for: Makorin 1 controls embryonic patterning by alleviating Bruno1-mediated repression of oskar translation
Source: PLoS Genet. 2020 Jan 24;16(1):e1008581. doi: 10.1371/journal.pgen.1008581 (PMC7001992; doi:10.1371/journal.pgen.1008581)
Supplement: S8 Table — (DOCX) [file pgen.1008581.s021.docx]

**S8 Table.** List of primers used to clone *osk* and *grk* reporter genes and introduce the mutations analyzed.

| **Name** | **Sequence** |
| --- | --- |
| osk_KpnI_F | AAAAGGTACCGGATCACTTTCCTCCAAGC |
| osk_SalI_R | AAAAGTCGACTAATGCAAGGTTGGAAACTG |
| osk_3UTR_EcoRV_F | AAAAGATATCGTTGGGTTCTTAATCAAGAT |
| osk_3UTR_SalI_R | AAAAGTCGACTAGCAAAGATTCAAGCCAAT |
| grk_3UTR_EcoRI_F | AAAAGAATTCGATTTAGAATTTGATTTGGA |
| grk_3UTR_SalI_R | AAAAGTCGACAAATGATGAAAGTGAGGAGA |
| osk_delta_Mkrn1_F | AATTGTATGTATTGATGGTGCAAGCTGCAATGTAAAATCC |
| osk_delta_Mkrn1_R | GGATTTTACATTGCAGCTTGCACCATCAATACATACAATT |
| osk_delta_AR_F | TTTGTCCTATAACAAGCTGCAATAAAAGGGAAATCAATGA |
| osk_delta_AR_R | TCATTGATTTCCCTTTTATTGCAGCTTGTTATAGGACAAA |
